# Surface Laplacian of interfacial thermochemical potential: its role in solid-liquid pattern formation

**DOI:** 10.1038/s41526-021-00168-2

**Published:** 2021-11-02

**Authors:** Martin E. Glicksman, Peichen Wu, Kumar Ankit

**Affiliations:** 1 University of Florida, Department of Materials Science and Engineering, 100 Rhines Hall, Gainesville, FL 32611-6400 USA; 2 Professor Emeritus, Florida Institute of Technology, Melbourne, Fl 32901 USA; 3 Materials Science and Engineering, School for Engineering of Matter, Transport, and Energy, Arizona State University, 551 E. Tyler Mall, Tempe, AZ 85287-6106 USA

**Keywords:** Physics, Theory and computation

## Abstract

Steady-state solid-liquid interfaces allow both analytic description as sharp-interface profiles, and numerical simulation via phase-field modeling as stationary diffuse-interface microstructures. Profiles for sharp interfaces reveal their exact shapes and allow identification of the thermodynamic origin of all interfacial capillary fields, including distributions of curvature, thermochemical potential, gradients, fluxes, and surface Laplacians. By contrast, simulated diffuse interface images allow thermodynamic evolution and measurement of interfacial temperatures and fluxes. Quantitative results using both approaches verify these capillary fields and their divergent heat flow, to provide insights into interface energy balances, dynamic pattern formation, and novel methods for microstructure control. The microgravity environment of low-Earth orbit was proven useful in past studies of solidification phenomena. We suggest that NASA’s ISS National Lab can uniquely accommodate aspects of experimental research needed to explore these novel topics.

## Introduction

Over the past decade, one of the present authors published studies applying field-theoretic methods to identify and quantitate interfacial thermodynamic fields^1,2^. Traditional solidification analyses of pure materials and alloys employ the energy and species assays when analyzing solid-liquid transformations that rely on "Stefan balances”^3^. Stefan balances directly relate interfacial growth speed to first-order energy and solute exchanges. Stefan balances, consequently, overlook higher-order energy and solute contributions that subtly derive from the higher-order actions of capillary forces, and represent small, but significant, deterministic interface perturbations that affect solidification kinetics.

Along with several other investigations, e.g.,^4,5^, the present authors developed precision post-processing numerical methods that analyzed and measured interfacial parameters using multiphase-field simulations of selected *stationary* microstructures. Steady-state simulations allow measurement of interfacial temperature fields along curved diffuse interfaces, described numerically by "isolines” of their paired phase-field indices^6–8^. Results from those studies, when compared with predictions derived from variational analyses of similarly shaped, but perfectly sharp, solid-liquid (*s*/*ℓ*) profiles, led to understanding and appreciation of how capillary-mediated interfacial energy sources and sinks arise deterministically, modify interface energy balances in pure systems, and ultimately influence interface motion and pattern-formation dynamics.

Hard experimental evidence, however, still eludes us, and is needed to elucidate interface capillary effects, and to support the exploration of follow-on efforts to control them in practical solidification and crystal growth processes. How, for example, would one determine whether or not capillary-mediated interfacial energy exchanges and species transfer—in addition to just random noise^9,10^—impart significant influence on interface dynamics, stability, and pattern formation? The present absence of needed experimental evidence is due to as yet unmet challenges in the experimental measurements needed to probe minuscule milli-Kelvin temperature variations occurring over micrometer interfacial length scales, and to accomplish these difficult measurements on moving interfaces at their melting temperature.

The purpose of this paper is to provide a wider awareness of higher-order interfacial capillary effects to researchers interested in exploring further uses of the microgravity environment for basic solidification research aboard NASA’s ISS-National Laboratory. Theory and new examples are provided herein to demonstrate the presence of deterministic—and thus controllable—capillary fields that act on selected two-phase microstructures in pure crystal-melt systems.

The now classical analyses of solid-liquid interfacial stability, by Mullins and Sekerka, and, independently, that by Voronkov, describe linear dynamics of random perturbations acting on planar interfaces and spherical precipitates^11–13^. The basis functions with which these investigators chose to represent "random” interfacial disturbances mathematically along isotropic planar *s*/*ℓ* interfaces were 2-D sinusoids, with waveforms described as $y=\delta \sin {k}_{x}x$. Here *x* and *y* are a perturbation’s physical coordinates; *δ* is the wave’s *y*-amplitude; and *k*_*x*_ = 2*π*/*λ* is an arbitrary wavenumber [m^−1^], the value of which is the number of cycles (waves) per unit distance in the *x*-direction. The wavenumber admits all possible wavelengths, *λ*, from near zero, up to the size of the planar solidification front. Their choice of a "spectrum” of sinusoidal perturbations underscores their *stochastic* description of interfacial disturbances—now a well-understood approach, and widely accepted by the solidification and crystal growth communities. Most notably perhaps, linear stability theory in its long-wave limit, captures and integrates all the results of constitutional supercooling theory, which was the prior accepted stability paradigm for *s*/*ℓ* interfaces of the 1950s^14^.

More than a century ago, however, Henry Poincaré cautioned about assigning randomness to "explain” ostensibly chaotic events observed in physics and elsewhere, stating that, *A very small cause that escapes our notice determines a considerable effect that we cannot fail to see, and then we say that the effect is due to chance.* (Science et Méthode,1908). Interfacial instability and microstructure pattern formation, in our opinion, remain no exceptions to Poincaré’s prescient warning about randomness.

Fifty years ago, however, it was already demonstrated by direct observations^15^ that planar interfaces can spontaneously and reproducibly initiate *deterministic*, not random, instabilities and patterns. Specifically, interfacial instabilities in dilute alloys and nominally pure melts evolve into cells and then dendrites—ubiquitous *s*/*ℓ* microstructures found in metal ingots and castings, which were described two decades earlier by Bruce Chalmers and his co-workers^16,17^. The sequence of instabilities observed on otherwise featureless *s*/*ℓ* interfaces always occurred where grain boundaries, sub-boundaries, or even single dislocations, intersected the interface. Deterministic instabilities amplified around grain boundary intersections into 2-D single and double ridges, which then split into periodic chains of 3-D hillocks. Isolated single and double rings also reproducibly formed where individual dislocations intersected the *s*/*ℓ* interface. In regions that lacked significant substructure, a more chaotic surface pattern gradually evolved, which encroached from remote edge-meniscuses that always surround *s*/*ℓ* interfaces where they contact their confining crucible walls.

More recently, Shang and co-workers used phase-field methods to conclude, *that the underlying mechanism for dendritic side branching was deterministic rather than stochastic,* and that anisotropy and curvature, not noise, were the most important factors determining detailed microstructure^18^. It was not known, until recently, whether deterministic interfacial instabilities arose from geometric enhancements of heat conduction and local impurity diffusion, or from more subtle causes to be discussed in detail in this overview.

We briefly use sine waves again as exemplar interfacial profiles, to demonstrate how curvature induces controlled non-linearity. As Nobelist Frank Wilczck so aptly and succinctly stated it, *curvature participates in its own creation.*^19^. Wilczck specifically addressed the properties of 4-D space-time, but his lapidary statement remains valid for general thermodynamic systems, including interfaces with capillarity. Curvature, in fact, is the universally controlling property of manifolds in 3-D and 2-D that evolve in time.

It is convenient to introduce scale-free Cartesian coordinates, (*μ* = *x*/2Λ, *η* = *y*/2Λ), to describe non-dimensional *s*/*ℓ* sinusoidal interfaces, as well as other less familiar periodic profiles to be introduced later. A scale-free sine wave, for example, with arbitrary dimensionless amplitude *a* = *δ*/2Λ and dimensionless wave length, $\hat{\lambda }=\lambda /2{{\Lambda }}$, may be expressed as the function $\eta =a\sin {\hat{k}}_{\mu }\mu$: Physical distances implicit in its wavenumber, *k*_*x*_ = 2*π*/*λ* [m^−1^], wavelength, *λ* [m], and wave curvature, *κ* [m^−1^], all vanish by dividing or multiplying, respectively, by the "characteristic” length, 2Λ [m]. This reference length, Λ, is the thermo-capillary distance derived from the Euler-Lagrange equations in variational calculus, that are solved to find *s*/*ℓ* interfacial profiles with minimum energy^8,20^, namely,

1 $${{\Lambda }}\equiv \sqrt{\frac{{\gamma }_{s\ell {{\Omega }}}}{G{{\Delta }}{S}_{f}}}.$$

The length Λ is defined in Eq. (1) using the following parameters: *γ*_*sℓ*_ [J/m^2^] is the interface’s solid-liquid energy density; Ω [m^3^/mol] is the molar volume of the phases; *G* [K/m] is the temperature gradient that surrounds the *s*/*ℓ* interface; and Δ*S*_*f*_ [J/mol-K] is the system’s molar entropy of fusion.

An interface’s dimensionless wave-like profile is defined using the standard Cartesian form for sine waves. This profile, moreover, has a dimensionless fundamental wavenumber, ${\hat{k}}_{\mu }\equiv 2\pi /\hat{\lambda }$ chosen here as unity; with local dimensionless curvature, $\hat{\kappa }(\mu (\eta );a,{\hat{k}}_{\mu })$, expressed as the following function of *μ*,^21^,

2 $$\hat{\kappa }(\mu (\eta );a)=\frac{a\sin \mu }{{(1+{a}^{2}{\cos }^{2}\mu )}^{3/2}},\,\,\left({\hat{k}}_{\mu }=1\right).$$

The wave curvature, defined in Eq. (2), is positive for convex portions of a curved *s*/*ℓ* interface, where the center of the interface’s osculating circle resides in the solid phase, and is negative for concave regions, where the center of the interface’s osculating circle is located in the liquid phase.

**Fig. 1:**
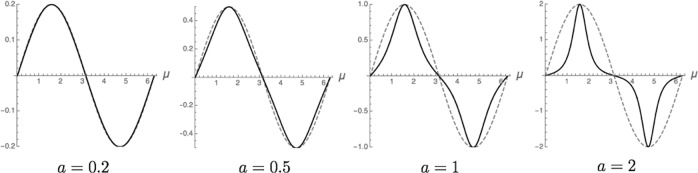
Sine wave curvatures and capillary non-linearity. Comparison of dimensionless in-plane curvatures [solid curves from Eq. (2)] shown with their profiles (broken curves). Profiles are plotted non-isometrically. Note the scale change on the ordinates with increasing amplitude, *a* Higher harmonic content of the curvature distribution is nearly absent at low wave amplitudes, *a* < 0.5, but increases rapidly as wave amplitudes exceed ca. *a* > 0.5.

Plotted (non-isometrically) in Fig. 1 are profiles of this sine wave, *μ*(*η*), along with their amplitude-dependent curvatures, $\hat{\kappa }(\mu (\eta );a)$, as calculated from Eq. (2). These pairs of curves demonstrate how local curvature (solid curves) increasingly deviates and becomes non-congruent with respect to its defining sine wave profile (broken curves) as the wave amplitude, *a*, increases. One finds that for amplitude *a* = 0.2, the dimensionless curvature is virtually indistinguishable—almost congruent—with the plotted profile that generates it. For *a* = 0.5, the curvature and generating profile deviate slightly. But at somewhat larger amplitudes, e.g., *a* = 1 and *a* = 2, curvature rapidly gains higher harmonic content and deviates from its generating profile.

Given that capillary forces on interfaces act in proportion to the local curvature, their importance, and thermodynamic influence increase rapidly, and non-linearly, as an interface departs from flatness. Indeed, one does find as Wilczck claimed that *curvature participates in its own creation.* The sensitive amplitude-dependence of curvature on shape becomes causal for deterministic self-interactions that spontaneously arise on curved interfaces, even, as will be demonstrated, on *stationary* interfaces. Also to be shown in this study, capillarity mediates pattern-formation on moving interfaces, adding determinism to microstructure formation.

Thermodynamic equilibrium at curved interfaces was first elucidated by Lord Kelvin (William Thomson)^22^. Thomson showed that interfaces, such as those found on droplets that separate a liquid from its vapor, with increasing curvature will experience an elevation in their chemical potentials at constant temperature, as reflected by an exponential rise in equilibrium vapor pressure. The so-named Kelvin effect explains fundamentally why fogs and droplet dispersions self-clear, and how clouds produce rain. In solid materials, Kelvin’s effect similarly explains why precipitate dispersions used to strengthen alloys become unstable, and coarsen at elevated temperatures.

Corresponding equilibria for curved interfaces between two *condensed* phases, such as a crystal and its melt at constant pressure, a situation where both phase densities change very little with temperature and pressure, may also be described with a linearization of Kelvin’s original exponential equation for fluids. The influence of curvature on *s*/*ℓ* equilibria is credited to J.W. Gibbs, and is known as the Gibbs-Thomson effect^23,24^. Note, that in the case of convex *s*/*ℓ* interfaces (positive curvature), the Gibbs-Thomson effect predicts a similar—but linear—increase in the interface’s thermochemical potential, as evidenced by a corresponding linear decrease in the interface’s equilibrium temperature, *T*_*int*_. In contrast, concave *s*/*ℓ* interfaces (negative curvature) lower their chemical potentials and raise their equilibrium temperatures.

Kelvin’s thermodynamic rules, as applied to *s*/*ℓ* interfacial equilibria, are captured by the Gibbs-Thomson equation that predicts the following linear change in the equilibrium interface temperature, *T*_int_, caused by local curvature.

3 $${T}_{\mathrm{int}}(y(x))-{T}_{m}=-\left(\frac{{\gamma }_{s\ell }\,{{\Omega }}}{{{\Delta }}{S}_{f}}\right)\kappa (x,y),\,\,\,({P}_{\mathrm{melt}}=\mathrm{const}.).$$

The lumped materials parameters within parenthesis on the right-hand side of eq. (3) collectively have SI units of [K ⋅ m]. Their product with an interface’s curvature, *κ*(*x*, *y*) [m^−1^], equals the local shift in *T*_int_ [K] caused by capillarity. For typical microstructures formed in many *s*/*ℓ* systems, these temperature shifts amount to ±10^−4^ to ±10^−2^ [K], which typically constitute the rather tiny range of thermocapillary effects that are usually ignored or simply overlooked.

However, if one also considers the small distances within a *s*/*ℓ* microstructure over which the curvatures and their Gibbs-Thomson temperatures fluctuate, or even change sign—recall the sine-wave case discussed previously—one finds that these microstructure distances vary from about a millimeter to several nanometers. This suggests that local *gradients* of the Gibbs-Thomson temperature, due to capillarity, occur along curved *s*/*ℓ* interfaces, from place-to-place and from time-to-time. These mesoscopic interfacial gradients have magnitudes from about 1 to 10^7^ [K/m], which are not at all inconsequential vector fields. Their influence, to be shown, has consequences.

Dividing through Eq. (3) by a "characteristic” thermo-capillary temperature interval equal to 2Λ*G* [K], where *G* is the local macrogradient in which a *s*/*ℓ* microstructure is embedded, yields a dimension-free equilibrium temperature change, or shift in thermochemical potential, that is induced locally by interfacial curvature at constant pressure, namely,

4 $$\frac{{T}_{\mathrm{int}}(y(x))-{T}_{m}}{2{{\Lambda }}G}=-\frac{1}{2{{\Lambda }}G}\left(\frac{{\gamma }_{s\ell }\,{{\Omega }}}{{{\Delta }}{S}_{f}}\right)\kappa (x,y).$$

Replacing the interface curvature, *κ*(*x*, *y*) [m^−1^], in Eq. (4) by its dimensionless form, namely, $\hat{\kappa }(\mu ,\eta )\equiv \kappa (x,y)\times 2{{\Lambda }}$, and again dividing the right-hand side by 2Λ, one obtains the dimensionless Gibbs-Thomson temperature shift, *ζ*_*int*_(*μ*, *η*). This shift is a scalar chemical potential field, *ζ*_*int*_(*μ*, *η*), found along any smooth *s*/*ℓ* interface, *μ*(*η*), with locally varying curvatures, $\hat{\kappa }(\mu ,\eta )$,

5 $${\zeta }_{\mathrm{int}}(\mu ,\eta )=-\frac{1}{4{{{\Lambda }}}^{2}}\left(\frac{{\gamma }_{s\ell }\,{{\Omega }}}{G{{\Delta }}{S}_{f}}\right)\hat{\kappa }(\mu ,\eta ).$$

Equation (5) simplifies further by dint of the consistent use of 2Λ as the system’s variational scaling distance. Specifically, by gathering all the lumped constants that defined Λ in Eq. (1), substituting them for Λ^2^ in Eq. (5), and then canceling terms, one obtains the thermochemical potential,

6 $${\zeta }_{\mathrm{int}}(\mu ,\eta )=-\frac{1}{4}\hat{\kappa }(\eta (\mu )).$$

Portrayal of the Gibbs-Thomson effect through Eq. (6) shows clearly that the distributions of curvature and the *ζ*_int_-potential represent proportionate geometric and thermodynamic fields. Modulo the pre-factor of $-\frac{1}{4}$, one finds that interfacial thermochemical potential equals its scaled curvature.

As shown next, the interface’s thermochemical potential field leads to other higher-order interfacial fields, including superficial vector gradients and their implied interfacial fluxes^25,26^. Furthermore, scalar divergences of those vector fields can lead to yet higher-order surface Laplacian sources and sinks, which can influence interface stability and affect dynamic pattern formation.

Adjustments in local equilibria, required by changes in the interface curvature as a *s*/*ℓ* microstructure evolves, occur at rates that are extremely fast. Establishing local equilibrium is fast because temperatures are close to the melting point, and the distances over which changes occur are microscopically small. The time-scales associated with local curvature changes, that adjust the interfacial distribution of the thermochemical potential, equals the squared thickness of the *s*/*ℓ* interface divided by the mean thermal, or solute, diffusivity. Our concern and distinction between sharp and diffuse interphase interfaces—which are the essential issues in this study—go back to Gibbsian thermodynamics, where an interface between two phases, A and B, each considered as a structureless continuum, was described as a "dividing surface” with zero thickness. For a one-component system, just the energy per unit area of the Gibbs dividing surface, *γ*_*AB*_ [J/m^2^], adequately serves to characterize an isotropic *sharp* interface.

One, of course, recognizes in the present study of stationary *s*/*ℓ* interfaces that real interphase interfaces must always have the added feature of a thickness. The reason is that solids—specifically crystalline phases—retain near-perfect long-range atomic/molecular order, even up to their melting points. By contrast, liquids, including melt phases, possess only limited short-range order, induced by short-range interactions among their randomly positioned and non-oriented atoms or molecules. Thus, *s*/*ℓ* interfaces *always* require a finite transition zone, or thickness, to separate bulk "ordered solid” from bulk "disordered liquid”. The limit of a perfectly sharp *s*/*ℓ* interfaces is indeed a fiction. Typical transitions required between crystalline solids and their liquid phases are estimated to be as small as several atomic diameters, and up to ten times that distance.

## Methods

### Study overview

Of particular interest in this study is the use of a class of non-planar *s*/*ℓ* interfaces that can be held stationary for an indefinite period, by applying a steady, uniform, temperature gradient. Note, that such stationary interfaces differ greatly from classic equilibrium Wulff shapes^27,28^, which are instead isothermal crystalline objects that achieve full thermodynamic equilibrium. Wulff shapes, in contrast with the constrained steady-state interfaces of interest here, are isothermal, closed equilibrium bodies. They exhibit spatially uniform chemical potentials that lack gradients, and adopt optimal shapes which minimize their total energy. The stationary microstructures of interest here are non-equilibrium systems constrained at steady-state by entropy-producing thermal gradients.

**Fig. 2:**
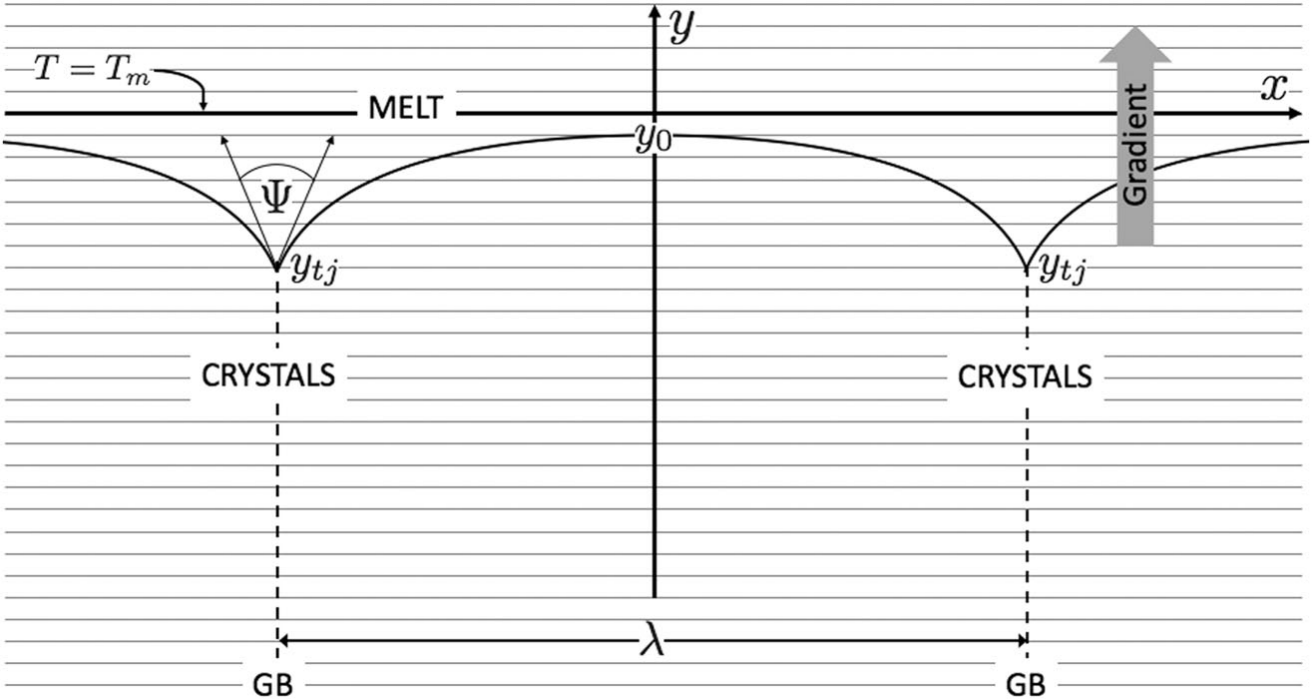
Grain boundary grooves, GBGs, linked along a stationary curved *s*/*ℓ* interface. Periodic GBGs separate bulk crystal and melt that support energy flow through a constant thermal gradient, pointing in the +*y* direction. The thermal field constraining the *s*/*ℓ* interface is illustrated here by a uniform grid of isotherms, where the *x*-axis coincides with the system’s melting-point, *T*_*m*_. Triple junctions formed at each grain boundary (GB) intersection are spaced apart a distance *λ* [m] along the *x*-axis. All triple junctions are located on coordinate *y*_*tj*_. Each triple junction supports a constant dihedral angle, 0 ≤ Ψ < *π*. Midpoints of the GBGs are located periodically at coordinate *y*_0_ < 0, where the slope is zero, and curvature exists.

Figure 2 shows an example of the stationary, curved *s*/*ℓ* interface that was used in this study. Its microstructure consists of linked GBGs. These grooves are formed by parallel grain boundaries (GB) that periodically intersect a stationary *s*/*ℓ* interface, which originally colocated with the *x*-axis. The *x*-axis corresponds with the one-component system’s melting point isotherm, *T* = *T*_*m*_. The resulting profile at steady-state separates pure solid from its melt, each phase accorded identical thermal conductivity and molar volume.

This stationary microstructure supports continuous heat-flow throughout the bulk phases, thus precluding full thermodynamic equilibrium. Only *local equilibrium* is allowed, which is limited to the *s*/*ℓ* interface itself. The applied temperature field that constrains this microstructure is illustrated in Fig. 2. This field, *T*(*y*), is represented by parallel isotherms with an arbitrary, but uniform, gradient, of magnitude *dT*/*dy* = *G* [K/m], pointing in the +*y* direction.

The curved interfaces abruptly separate two bulk phases. The solid and liquid that surround each GBG support steady heat flow, and produce entropy continuously over the entire (*x*, *y*)-plane. The *s*/*ℓ* interface occupies a relatively narrow 2-D region within quadrants III and IV of the (*x*, *y*)-coordinate system, where the coordinates of the triple junctions are *y*_*tj* _≤ *y*(*x*) ≤ *y*_0_ < 0. Recall that *only* the *s*/*ℓ* interface achieves local equilibrium, as required by the Gibbs-Thomson effect and its Eq. (6). The Gibbs-Thomson effect matches the chemical potentials of the two phases and equilibrates them (locally) along their curved interfaces. The balance of this *s*/*ℓ* system remains locked into steady-state: immobile, unchanging, and continuously producing entropy everywhere.

Single, as well as multiple, GBGs appear spontaneously on *s*/*ℓ* interfaces. Such *s*/*ℓ* microstructures form sometimes by a single or nearly isolated grain boundary. Bolling and Tiller^29^ first determined the steady-state profile for such isolated GBGs with equal thermal conductivity and molar volume of the solid and liquid, constrained by a uniform thermal gradient. More general GBGs formed between one-component phases with markedly different thermal conductivity, such as occur in water-ice interfaces^30^, are consequently constrained by more complex, non-uniform steady thermal fields, which were analyzed by Nash and Glicksman^31^. GBGs are frequently employed experimentally to measure average solid-liquid energies for many different *s*/*ℓ* systems^32,33–37^, and studied dynamically for their effect on interfacial stability during solidification^38,39^.

## Results and discussion

### Sharp-interface profiles

The present authors determined the mathematical solution for the steady-state variational profiles of periodic GBGs with equal phase conductivities and molar volumes, as depicted in Fig. 2. In the interest of brevity, only our final analytical results are presented here for these periodic GBG profiles. Detailed methods needed to derive the following variational solution and the determination of thermodynamic free energies for GBG formation will be published elsewhere. We will concentrate on their use.

The (*μ*, *η*) coordinate system needed to describe such periodic GBG profiles was presented in the *Introduction*. The (mirror-image) semi-profiles of periodic GBGs, *μ* = ±U(*η*; *η*_0_, Ψ), are defined by the following equation pair:

7 $$\begin{array}{rcl}&&\pm {{{\rm{U}}}}(\eta ;{\eta }_{0},{{\Psi }})\,=\,\,\frac{\left(2{\eta }_{0}^{2}+1\right)}{2{\eta }_{0}}{{{\rm{Elliptic}}}}\,{{{\rm{F}}}}\left[\frac{1}{2}\,\arccos \,\left(1\,-\,2\,\left({\eta }^{2}\,-\,{\eta }_{0}^{2}\right)\,\right)\,,\,-\frac{1}{{\eta }_{0}^{2}}\right]\\ &&-{\eta }_{0}\,{{{\rm{Elliptic}}}}\,{{{\rm{E}}}}\left[\frac{1}{2}\,\arccos \,\left(1\,-\,2\,\left({\eta }^{2}-{\eta }_{0}^{2}\right)\,\right)\,,-\frac{1}{{\eta }_{0}^{2}}\right],\,\,({\eta }_{tj}\le {\eta }_{0} \,<\, 0).\end{array}$$

Unit profiles for periodic GBGs begin and end at their triple junctions, each located at the profile’s most negative *η*-coordinate, ${\eta }_{tj}=-\sqrt{{\eta }_{0}^{2}-\frac{1}{2}\left(\sin \,\left(\frac{{{\Psi }}}{2}\right)\right.\,+\,\frac{1}{2}}.$ Note that the ordinate of the triple junctions, *η*_*tj*_, as does the entire profile, fundamentally depends on two parameters: the midpoint, *η*_0_, and the dihedral angle, Ψ.

Equation (7) is cast here as weighted incomplete elliptic integrals of the first and second kind. We use the formal nomenclature of Gradshteyn and Reyzhik^40^. The restriction that sets the range of the independent *η*-variable in Eq. (7) requires specification of the GBG’s dihedral angle, 0 ≤ Ψ < *π*. In addition, incomplete elliptic integrals, such as Elliptic E and Elliptic F, require an "angular-amplitude", expressed here as the term in brackets before the functions’ commas. This amplitude depends both on the running variable *η*, and a GBG profile’s midpoint parameter, *η*_0_. Incomplete elliptic integrals also require a second, independent "*m*-parameter”, given here as the negative term in brackets after the functions’ commas. The *m*-parameter depends only on a profile’s midpoint coordinate, *η*_0_, which is a constant always less than zero.

Using this nomenclature, one finds that each variational GBG profile requires specification of two key numbers: (1) the value of the profile’s midpoint coordinate, *η*_0_ < 0, always located below the system’s melting point isotherm (*η* = 0), and (2) the value of the profile’s steady-state dihedral angle, Ψ. The Ψ-value affects the interface profile by limiting the eigenrange of the profile’s slopes. Slopes vary between a profile’s midpoint value, *η*_0_, where by symmetry the slope is set to zero, and its triple junctions, *η*_*tj*_, where the slope angles are respectively ±(*π* − Ψ)/2. See again a GBG’s configuration as illustrated in Fig. 2.

Surprisingly, these formal spatially periodic profile formulae do not require input of a grain boundary spacing, which, in nature, provides the physically causal feature that sets the form and spacing of these common microstructures. As discussed later, a specified grain boundary spacing is always required for phase-field simulation of a GBG, but is not needed to calculate their profiles from variational theory. Instead, the elliptic integral solution, Eq. (7), determines a self-consistent sharp-interface profile for any combination of dihedral angle, Ψ, and midpoint parameter, *η*_0_ < 0. The steady-state separation of their triple junctions, located at *η* = *η*_*tj*_, is equivalent to the more realistic presence of periodically spaced grain boundaries. GBG variational profiles are made periodic by joining their semi-profiles at their common midpoint, *η*_0_, and then linking those units along the *μ*-direction.

Although not obvious in the Cartesian setting given in Eq. (7), the angular amplitude of these elliptic integrals equals the half-angle of the profile’s slopes. Slopes, as mentioned above, are limited in magnitude by the profile’s dihedral angle. In sum, a GBG’s stationary profile, according to variational theory, requires evaluation of two-parameter functions, viz., the incomplete elliptic integrals. These functions include: (1) the profile’s midpoint ordinate, *η*_0_, a number less than zero, and (2) the profile’s dihedral angle, 0 ≤ Ψ < *π*. This parameter pair completely determines the periodic GBG’s profile. The GBG’s shape, repeat spacing, and location relative to the system’s applied thermal field establishes the coordinate location for *ζ*_*int*_ = 0, where one sets *η* ≡ 0 > *η*_0_. All these geometric and thermodynamic features may be reproduced and verified by directly comparing their exact analytic shapes, viz., Eq. (7), with counterpart images independently simulated with a multiphase-field model.

**Fig. 3:**
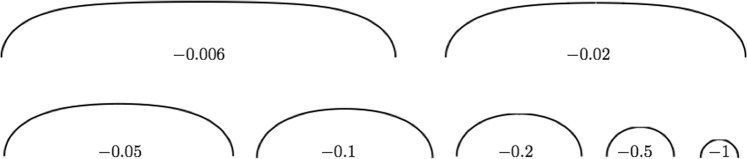
Profiles of sharp-interface GBGs, plotted from eq. (7), with zero dihedral angle. Each steady-state profile is labeled by its *η*_0_-value. The value *η*_0_ equals the vertical distance between the profile’s midpoint and the system’s isopotential *ζ*_*int*_ = 0. As *η*_0_ → 0, the profile width, or grain boundary separation, diverges, and its midpoint region approaches true flatness. The limiting periodic GBG configuration (*η*_0_ = 0) formally corresponds to the class of "isolated GBGs'', for which a profile formula consisting of elementary functions was first derived by Bolling and Tiller^29^.

The advantage of analyzing steady-state sharp-interface profiles is that their variational shapes satisfy a non-linear ordinary differential equation (ODE), which stipulates that local interface potential equals local interface curvature, modulo a constant of proportionality. Interfacial capillary-mediated fields are then easily calculated from these *analytically* defined sharp profiles. Most importantly, however, using field theory the origin and characteristics of capillary-mediated scalar and vector fields are precisely identified from a thermodynamic standpoint, and their interfacial distributions determined^8,41^. A sampling of steady-state, sharp-interface GBG profiles, derived from Eq. (7) is shown in Fig. 3 for several midpoint values, *η*_0_.

### Diffuse-interface microstructures

To achieve independent and accurate comparisons with sharp-interface variational profiles, we simulated multiphase-field images of steady-state GBG microstructures. Phase-field images and their companion thermal fields were determined by numerically solving coupled partial differential equations (PDEs) through sequential, small, time steps that accurately portray a system’s *evolved* sequence of thermodynamic states. We emphasized that it was essential for the boundary conditions required by analytically derived sharp-interface GBG profiles be consistent with the same periodic environment as that used to simulate their diffuse-interface microstructures via phase-field modeling. Selecting consistent boundary conditions for both approaches allows close, meaningful comparisons between simulated phase-field images and measured potentials on diffuse-interface microstructures with predicted fields, the latter identified and calculated by analysis of their counterpart sharp-interface variational profiles.

Note, however, that the applied, linear thermal field, illustrated in the analytic model shown in Fig. 2, imposes, ab initio, a uniform temperature gradient on the *s*/*ℓ* profile. That ab initio gradient differs in detail from the evolved local potential gradients associated with the *simulated* temperature field that co-develops with each steady-state phase-field microstructure. When applying phase-field simulation, two temperatures and a calculation box distance are specified as inputs, instead of applying a uniform gradient. Specifically, a temperature above the melting point is selected as the upper limit of the phase-field computation box, and a second temperature below the melting point is selected as the lower-limit. Importantly, we learned that the thermal field that finally develops around a simulated microstructure includes *all* the system’s capillary-mediated thermodynamic fields. Not all these fields are admissible, however, in the solutions for the variational sharp-interface profile, for reasons to be discussed in detail. These subtle, but critical differences between variational profiles and simulated images will be elucidated later in this section.

Multiphase-field models also rely on continuum descriptions of matter, and are solved numerically from a system of simultaneous PDEs. Phase-field models solve for the evolving thermodynamic state that develops over time as two or more phases, labeled, say, as 0, 1, and 2. These phases evolve their collective space-time trajectories to form the microstructure. Phase-field models respect thermodynamic rules for the system’s energy and entropy, and obey the locality (impenetrability) and indestructible nature of matter. Such models produce reasonably realistic images of polyphase structures—here *s*/*ℓ* microstructures—that evolve dynamically over time. The two-phase microstructures that form here, are rather special; they all eventually relax into a steady-state, so these simulated systems never attain full thermodynamic equilibrium.

In brief, the images produced by simulation actually consist of *continuous* phase regions: two solid phases, labeled phases "0" and "1" that differ only in their crystallographic orientation, and their common equilibrium melt phase, labeled phase "2". Each equilibrium phase, *i* = 0, 1, or 2, is "fully present” when its phase index, *ϕ*_*i*_ = 1, and "completely absent” when *ϕ*_*i*_ = 0. One may also think of intermediate states where *ϕ*_*i*_ (0 < *ϕ*_*i*_ < 1) are lines—loci that act as avatars—for the *continuous* states of matter needed to bridge between adjacent bulk phases. Thus, inasmuch as *ϕ*_*i*_ = 1 or 0 denote, respectively, the presence, or absence, of equilibrium phase *i*, intermediate values of their phase indices represent the model’s higher energy states of matter that exist *between* pairs of bulk equilibrium phases to form their diffuse interfaces. This continuous variation of all the phase indices, from 0 to 1, over a single computational domain, underlies the essence of phase-field models. This scheme eliminates the daunting task of tracking the motions of distinct, sharp, "free boundaries”, yet allows complex geometric development of continuous phase domains, including even topological changes like pinch-off, nucleation, and particle vanishing, that can occur during microstructure evolution.

**Fig. 4:**
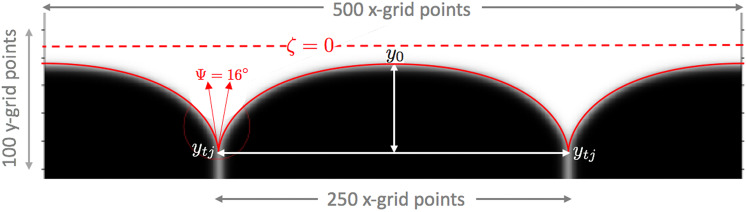
Comparison of a multiphase-field simulated image of a periodic GBG with its variational sharp-interface counterpart, the *μ*-*η* profile (red curve). To achieve the fit shown in the figure, uniform magnification was applied to the isometric *μ*-*η* profile, calculated with the same dihedral angle used in the simulation, viz., Ψ = 16 deg. The value of Ψ is the only parameter shared between models. The goodness of fit between the simulated GBG image and its sharp-interface profile is estimated from their respective aspect ratios, *A* and $\hat{A}$, where, *A* is measured on the phase-field image, and $\hat{A}$ is calculated from Eq. (7) for the variational profile. The *A*-value was *measured* using the white-arrow distances shown on this image, and a matching $\hat{A}$-value was *calculated* from Eq. (7) using iterated *η*_0_ values. *η*_0_ converged to −0.095, a value estimated to be accurate to three significant figures. The quality of the fit between models is limited by the pixel resolution of the interfacial isolines that define the phase-field image. The broken red line represents the theoretical position of the melting point isotherm, or isopotential, *ζ* = 0, where a flat *s*/*ℓ* interface would reach equilibrium.

Thus, simulated equilibrium phases are separated from each other by diffuse interfaces—actually a tight distribution of their *ϕ*_*i*_-index isolines—that describe the continuous spatial transitions between evolving phase regions. This "bridge", or transition between phases S_0_ ↔ S_1_, represents their intermediate "grain boundaries", whereas those between phases S_0_ ↔ L_2_, and S_1_ ↔ L_2_ represent their equivalent "*s*/*ℓ* interfaces". These diffuse interfaces actually provide more realistic evolving phase contours that also visually define the temporal and spatial aspects of a microstructure’s development. For example, Fig. 4 shows three interphase interfaces—actually three *ϕ*_*i*_-index contours—that meet at triple junctions spaced periodically between adjoining crystals and their adjacent melt phase. The left and right half-crystals shown in this image are mirror images, because the microstructure is specified through periodic boundary conditions identical to those applied to the sharp-interface variational profile described with Eq. (7).

To avoid improperly reducing the simulated system’s interfacial energy by just trivially reducing all interfaces to zero-thickness, i.e., again replacing the matter and excess energy associated with phase borders by an unrealistic sharp "dividing surface”, the gradients of the *ϕ*_*i*_-indices normal to their isolines for each phase pair are evaluated. Each gradient contributes a "penalty”, or energy cost, charged according to the local steepness of that gradient. The phase-field model accomplishes all this complex dynamic accounting and seeks an appropriate thermodynamic pathway, which requires large-scale fast computations that converge efficiently to resolve the finer details of the loci of all interphase isolines. Multiphase-field models now provide sufficiently realistic phase images that impart diffuseness to their interfaces, as appropriate to the pixel density and computational capacity of these simulations.

Figure 4 also shows the comparison achieved between a simulated image and its counterpart analytic profile for a periodic GBG with a 16 deg. dihedral angle. The grain boundary spacing, *λ*, chosen for the simulation was 250 x-grid units in the phase-field computation box. The phase-field image shown here developed its steady-state after several hundred-thousand computational steps, when the dihedral angle stabilized at its steady-state value of 16.0062 deg. within 1 part per thousand of the desired input value. The vertical distance between the simulated GBG image midpoints, *y*_0_, and its triple junctions, *y*_*tj*_, settled at 62 ± 0.1*y*-grid units. The measurements of depth-to-width equal the image’s *simulated* steady-state aspect ratio, viz., *A* ≡ (*y*_0_ − *y*_*tj*_)/*λ* = 0.248 ± 0.008.

Theoretical aspect ratios, $\hat{A}$, were then calculated for this phase-field image’s counterpart sharp-interface variational profile, where $\hat{A}$-values were obtained from Eq. (7), using a matching dihedral angle of Ψ = 16 deg. Iteration on the profile’s *η*_0_ midpoint value continued until the theoretical aspect ratio agreed with the mean value of the measured phase-field ratio: viz., $\hat{A}\,\,\,\ \iff \ \,\,\,A=\,0.248\pm 0.0005$. When the value of the sharp-interface aspect ratio, $\hat{A}$, agreed, within the measured tolerance, with the phase image aspect ratio, *A*, its iterated midpoint coordinate converged to *η*_0_ = −0.095 ± 0.001; the triple junction coordinate, *η*_*tj*_ = −0.663; and the profile’s periodic dimensionless spacing between triple junctions was Δ*μ* = 2.289.

These parameters collectively provide the statistical match shown in Fig. 4, between the theoretical sharp-interface profile, Eq. (7), and its simulated diffuse-interface image. Equation (7) provided (*μ*, *η*)-coordinates that determine the sharp-interface profile, from which a (red) variational profile curve was isometrically magnified and added to Fig. 4, to envelop the simulated steady-state image. The "goodness of fit” achieved between the phase image and the sharp-interface profile, shown in Fig. 4, is ±0.5%, a precision limited by the pixel resolution of the phase-field’s digital image, which controls the accuracy of its measured aspect ratio, *A*.

It is the diffuseness of a *s*/*ℓ* interface that determines its fundamental ability to transport energy or solute tangentially by tangential interfacial gradients of the capillary-mediated potential. Sharp (non-diffuse) interfaces, such as the variational profiles calculated from Eq. (7), are incapable of supporting any thermal conductance or species diffusion. Their surface transport number, specifically the interfacial thermal conductance, *k*_int_ [W/K], is zero in this extreme limit.

The issue of isoline pixel width, and its effective tangential thermal conductance of isoline image borders was investigated recently by the present authors. We measured the intensity of capillary-mediated fluxes induced by curvature gradients developed along steady-state phase-field images of GBGs with *s*/*ℓ* isoline borders having different diffusenesses, or pixel widths. Perhaps not too surprising, the effective tangential interfacial conductance along such GBGs increased linearly with their diffuseness^42^. Although that finding was both an interesting and satisfying result from the multiphase-field model, the subjects of capillary-mediated fields and the detailed influences of interface diffuseness still lack direct experimental probing. This remains a knowledge gap concerning interfacial structure that could be closed by well-controlled microgravity experiments.

### Curvature and potential gradients

The key idea offered from this study is that real, or simulated, diffuse-interface GBGs develop curvatures and capillary-mediated potential gradients, the scaled distributions of which are essentially *identical* to those calculated for their sharp-interface counterparts. These matched geometric and thermodynamic gradient fields, however, cause profoundly different effects: viz., gradients along diffuse interfaces stimulate tangential fluxes, whereas gradients along sharp-interface profiles do not.

The same scalar divergences that cause divergent vector flux fields to act as sources and sinks along diffuse interfaces exist only as proportional "inert divergences” of the gradient field along sharp-interface GBGs. This odd dichotomy in the behavior of the scalar divergence of the thermochemical gradient, or, to be shown next, the surface Laplacian of the thermochemical potential, is explained by the fact that perfectly sharp "Gibbsian interfaces” have zero tangential conductance, which, of course, precludes any flux response from the presence of interfacial potential gradients.

Specifically, *only* interfaces with steady-state curvature distributions support time-independent spatial distributions of their thermo-potential and curvatures. Equation (6)—a dimensionless form of the Gibbs-Thomson equation—relates curvature and thermochemical potential. The two restrictions needed to derive that linear relationship are those that also undergird the Gibbs-Thomson equation: (1) the interface is at *local* equilibrium, and (2) the interface is sharp. The questions of exactly how sharp or diffuse a *s*/*ℓ* interface must be to respond under these thermodynamic conditions, and to influence interface dynamics, are among the issues addressed in this research, and which will doubtless demand further study.

### Gradients of the potential

The tangential gradient of an interfacial scalar distribution is a vector field pointing along the interface’s (dimensionless) arc length, $\hat{s}\equiv s/2{{\Lambda }}$,^43^. The normal component of this gradient field remains zero everywhere along the interface. An interface’s arc length in (*μ*, *η*)-coordinates has the total differential $d\hat{s}=\sqrt{d{\mu }^{2}+d{\eta }^{2}}$, from which application of the chain rule, $\frac{d{\zeta }_{int}}{d\hat{s}}=\frac{d{\zeta }_{int}}{d\eta }\cdot \frac{d\eta }{d\hat{s}}$, yields the tangential, or arc-length vector gradient of the thermochemical potential, $d{\zeta }_{int}/d\hat{s}\cdot \overrightarrow{\tau }$. Here, $\overrightarrow{\tau }$ is the unit tangent vector along the interface. The unit tangent extends over the profile’s range of *η*-values indicated in Eq. (8) for a periodic GBG, and is given by the following vector equation,

8 $$\left(\frac{d{\zeta }_{int}}{d\hat{s}}\right)\cdot \overrightarrow{\tau }=\,-\frac{1}{4}\left[\left(\frac{d\hat{\kappa }}{d\eta }\right)\,\cdot \,\frac{1}{\sqrt{1+{(d\mu /d\eta )}^{2}}}\right]\,\cdot \,\overrightarrow{\tau },\,\,\,({\eta }_{tj}\le \eta \le {\eta }_{0}).$$

Carrying through the steps indicated in Eq. (8), and using Eq. (7) to evaluate $\hat{\kappa }$, $d\hat{\kappa }/d\eta$, and *dμ*/*dη*, one obtains the arc-length derivative, or tangential *τ*-gradient of the interfacial thermochemical potential along sharp-interface variational GBGs. The magnitude of the tangential potential gradient is independent of the dihedral angle. The dihedral angle, however, determines the spacing of the GBG’s triple points for a given midpoint value, *η*_0_, and thereby sets the allowed range of gradients developed along a GBG interface.

9 $${\overrightarrow{\nabla }}_{\tau }\left[{\zeta }_{\mathrm{int}}(\eta ;{\eta }_{0})\right]=-8\sqrt{\left({\eta }^{2}-{\eta }_{0}^{2}\right)-{\left(\eta -{\eta }_{0}\right)}^{2}{\left(\eta +{\eta }_{0}\right)}^{2}},\,\,\,({\eta }_{tj}\le \eta \le {\eta }_{0}).$$

**Fig. 5:**
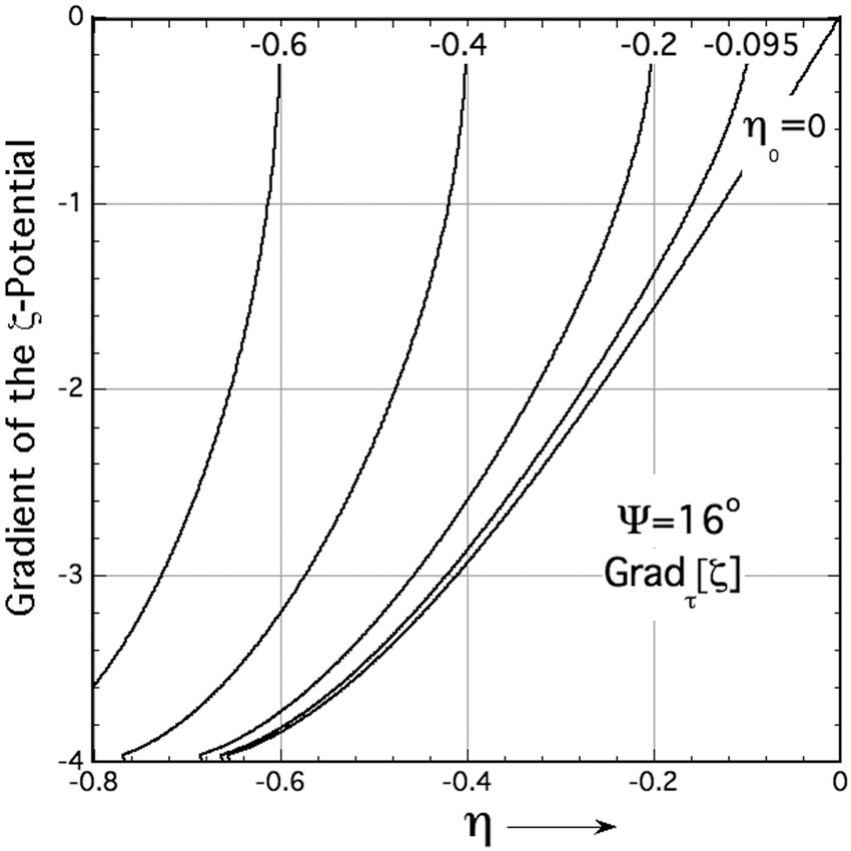
Plots of Eq. (9) showing tangential gradients of the interface for various GBGs with a dihedral angle of 16^∘^. Each curve represents a specific midpoint-value, *η*_0_, noted near its upper ordinate. The plot for *η*_0_ = −0.095 corresponds to the gradient associated with the red sharp-interface profile added in Fig. 4, whereas the plot for *η*_0_ = 0 shows the limiting behavior of the tangential gradients for an isolated GBG with the same dihedral angle. GBGs with Ψ = 16^∘^ develop their maximum gradient magnitudes of ≈4 at their differing triple junctions, *η*_*tj*_. Gradients all vanish, by symmetry, at a GBG’s midpoints.

Several plots of the capillary-induced gradient, Eq. (9), are shown in Fig. 5 for GBGs with various *η*_0_ values and a dihedral angle of Ψ =  16 deg. Sharp interface profiles of periodic GBGs support negative tangential vector gradients that point opposite to the profile’s unit tangent. By convention, tangent vectors point anti-clockwise along a *s*/*ℓ* interface, keeping solid always on the left. The plots in Fig. 5 show that for this range of midpoint values the most intense gradients tend to surround the GBG’s triple junctions. Their intensities diminish to zero at their midpoints, because of mirror symmetry applied at that point.

### Laplacian of the potential

The surface Laplacian of the thermochemical potential, ${\nabla }_{\tau }^{2}\left[{\zeta }_{int}(\eta ;{\eta }_{0})\right]$, equals the scalar divergence of the tangential potential gradient, viz., ${\overrightarrow{\nabla }}_{\tau }\cdot {\overrightarrow{\nabla }}_{\tau }\left[{\zeta }_{int}(\eta ;{\eta }_{0})\right]$. The negative of these quantities represent a scalar distribution of capillary-mediated cooling rates along a diffuse *s*/*ℓ* GBG interface. The GBG’s surface Laplacian of *ζ*_int_ is easily found along a sharp interface profile, as a cubic polynomial of the running variable, *η*, namely,

10 $${\overrightarrow{\nabla }}_{\tau }^{2}\left[{\zeta }_{\mathrm{int}}(\eta ;{\eta }_{0})\right]=-16\eta \left(-2{\eta }^{2}+2{\eta }_{0}^{2}+1\right),\,\,\,({\eta }_{tj}\le \eta \le {\eta }_{0}).$$

**Fig. 6:**
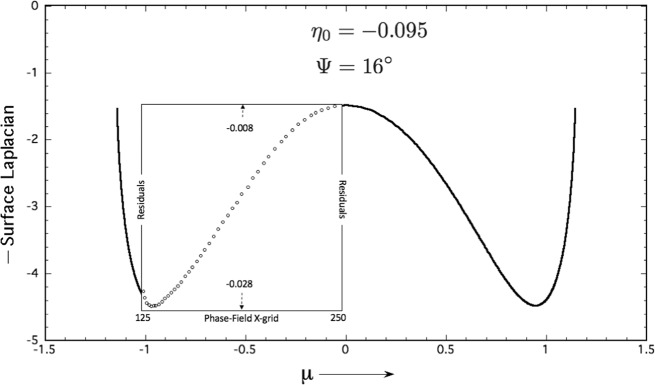
Minus the surface Laplacian of the thermochemical potential versus *μ*-coordinate along a sharp GBG interface profile (*η*_0_ = −0.095, Ψ =  16 deg). The boxed insert contains data for 46 scaled temperature residuals [K], which are proportionate to the local capillary-mediated cooling rate. Residuals were measured independently along the phase-field image isolines shown in Fig. 4. Residuals are the local isoline temperature minus the value of what a linear temperature field would be at that point, were the gradient a constant equal to the total temperature difference spread over the total *y*-grid, divided by the box height. The calculated surface Laplacian for the sharp interface is proportionate to the cooling rate developed along diffuse *s*/*ℓ* isolines that have identical divergences of their tangential heat flux. Distributed interfacial cooling is responsible for the development of non-linearities in the temperature field within the microstructure, as displayed in Fig. 7.

Figure 6 provides a cross-plot of the negative this surface Laplacian for the sharp-interface profile, *μ*(*η*) = U(*η*; *η*_0_, Ψ), calculated for the parameters Ψ = 16 deg, and midpoint, *η*_0_ = −0.095. Note again that although the surface Laplacian appears independent of the dihedral angle, the latter sets the triple point spacing, and limits the allowed range of the Laplacian. The same parameter pair was shown to specify a sharp-interface GBG, the shape of which is isometrically congruent with the diffuse-interface phase-field image shown in Fig. 4. The *μ*-distribution of these divergences over its range of potential gradients, plotted in Fig. 6, indicate an initial increase in the magnitude of the Laplacian, from its smallest magnitude of approximately −1.5 at the profile’s mid-point, to its largest magnitude of −4.5 approaching the profile’s triple junctions, where the Laplacian magnitude falls to −1.4 at each triple junction.

**Fig. 7:**
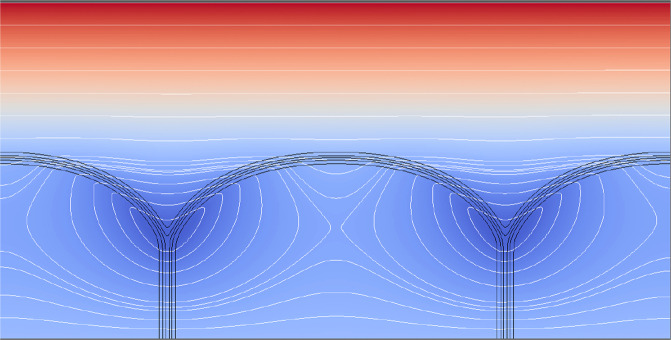
Steady-state temperature field (where red is warm, blue is cool) and its co-dependent phase-field image. Isotherms (white lines) remain nearly horizontal in the liquid phase, where they lie well above the *s*/*ℓ* (black) isolines. Lower isotherms break into loops surrounding the triple junctions, which are locations where capillary-mediated cooling, i.e., heat absorption, is most intense, forming cooler "blue valleys" that surround the triple junctions.

The steady-state phase-field images simulated in Figs. 4 and 7 have their profile shapes defined by dense isolines separating the bulk phases. This allows proportionate realization of their divergence distributions as *active* isoline cooling rates based on the surface Laplacian distribution shown in Fig. 6. These cooling rates were measured as proportionate to the depression of the thermochemical potential along the interface, and are plotted in Fig. 6.

### Residuals of the measured potential

**Fig. 8:**
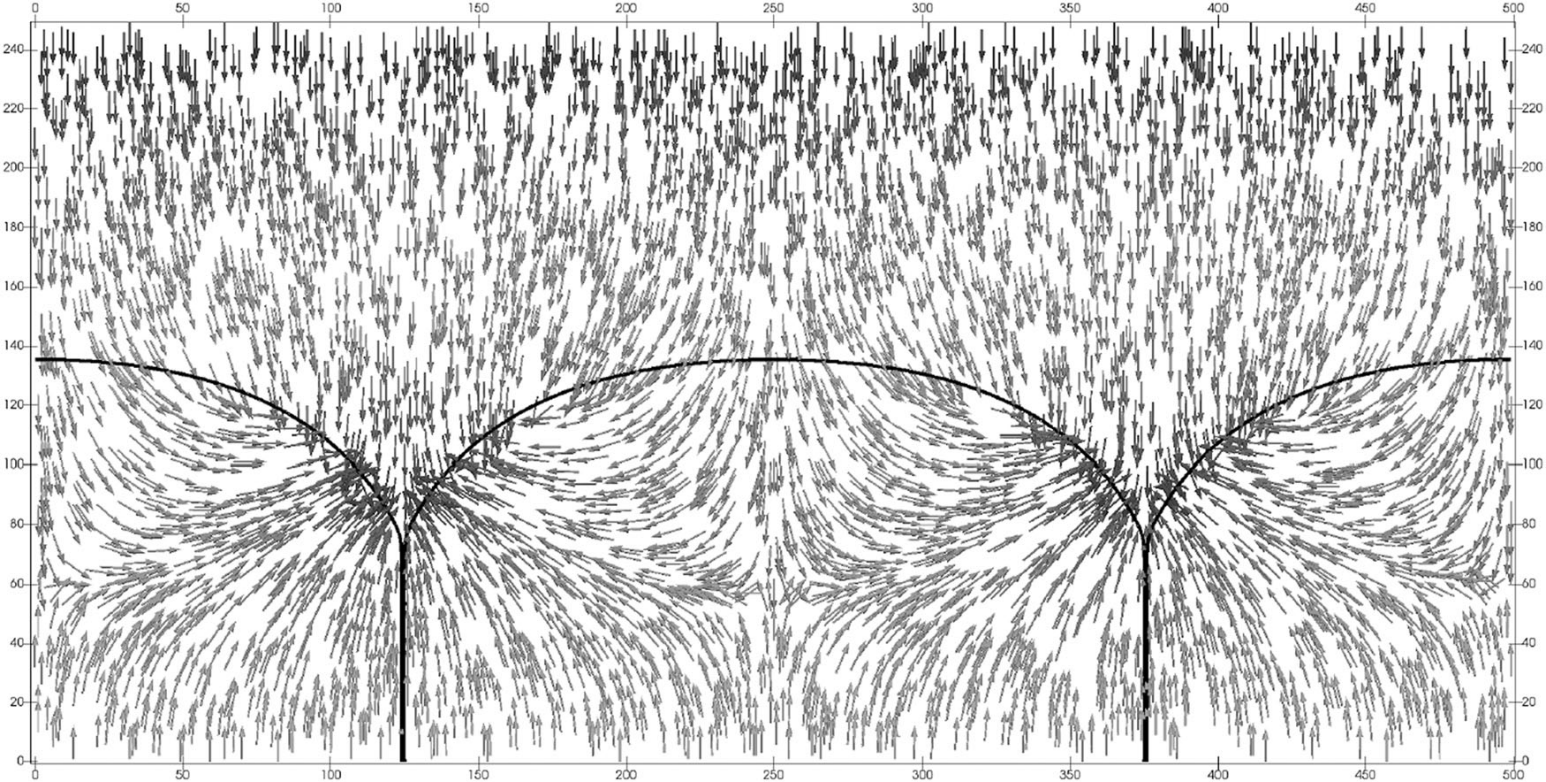
Vector heat-flow map for the steady-state periodic GBG microstructure in Figs. 4 and 7. The *s*/*ℓ* interfaces and their grain boundaries are shown as heavy black borders. Heat flow introduced through the hotter liquid has trajectories that remain downward above the stationary *s*/*ℓ* interface. Heat flow locally deflects toward the cooler "blue valleys", contoured on the thermal map shown in Fig. 7. Downward flow vectors, upon entering the solid, rapidly change their directions. Cooling rates become largest close to the triple junctions, and flux vectors terminate their pathways where they enter the interface. The flow map shows general "convergence'' of heat vectors associated with the applied temperature field toward capillary-cooled *s*/*ℓ* isolines, especially near the microstructure’s triple junctions.

Residuals data (depressions of the thermochemical potential from capillary cooling) reported in Fig. 6 were also measured with a post processing algorithm after steady state was achieved in the simulated phase-field image. These data closely follow the divergences of the tangential heat flux predicted from the surface Laplacian derived for the sharp-interface GBG profile. Interface potential reduction, called "residuals”, are direct manifestations of proportionate cooling rates experienced along the diffuse isolines of the simulated GBG. Laplacian cooling reduces the interfacial temperature (isoline chemical potential) below that expected from the applied thermal gradient acting alone. The imposed cooling rates by capillarity cause small, but measurable, milli-Kelvin depressions of the steady-state interface temperature, or thermochemical potential. Capillary-mediated cooling modifies the system’s temperature distribution, especially along and near the *s*/*ℓ* interface. Indeed, for the first time, the effect of capillary-mediated divergence and cooling can be directly visualized both on Fig. 7, the steady-state temperature distribution, and on Fig. 8, the corresponding heat-flux vector map.

To reiterate, the major assumption applied in our interpretation of the comparative behavior found between these geometrically related 2-phase microstructures is that we chose an analytic shape for the counterpart sharp-interface profile that has the same aspect ratio and profile as its diffuse-interface image. The former sharp interface profiles have, by virtue of their curvature distributions, identical vector gradients, but with *inert* divergences that lack the ability to cool the sharp interface. Diffuse interface images, by contrast, with similar curvatures, temperature gradients, and presumably comparable surface Laplacians of the chemical potential, permit *active* divergent heat fluxes that proportionately "cool” the phase image’s *s*/*ℓ* isolines, and probably cause very small, higher-order shape changes in the simulated profile, which at present remain below detectabilty.

### Steady-state divergences of the potential gradient

Verification of steady-state gradients in the Gibbs-Thomson potential along sharp-interface GBGs allow analytic examination of those vector fields to detect the presence of any scalar divergences. Divergences of *potential gradients* are proportional to the heat-flux divergences that they would actually impose if present on similar diffuse interfaces. Flux divergences on diffuse interfaces are proportional to the surface Laplacian calculated for the *ζ*_int_ potential along similarly profiled sharp-interfaces^26,44^. We plotted minus the surface Laplacian distribution in Fig. 6, the magnitudes of which are proportional to cooling rates expected, and to interface potential residuals actually measured along simulated, capillary-cooled, GBG microstructures.

### General divergences of chemical potential gradients

The reverse occurrences can happen too, on interfaces with distributions of curvature that present a negative surface Laplacian of the chemical potential, where "convergence” of the induced heat flux occurs. Convergent heat fluxes release heat and cause local heating. In the case of alloys, where solute species are present, they too would be rejected from the interface by the convergent tangential solute flow. GBGs do not exhibit negative surface Laplacians.

Finally, dynamic solidification shapes that develop during active freezing, including cells and dendrites, exhibit surface Laplacians with isolated roots, where interfacial convergent and divergent regions meet at isolated points along a moving interface. Near such Laplacian roots, interfacial bumps, or waves, are stimulated, that leads to deterministic branching and invagination of the interface^2,45^. These events tend to multiply over time and can lead to the development of extremely complex *s*/*ℓ* interfacial patterns.

### Conclusions

• An overview is provided of recent research into capillary-mediated thermodynamic fields on *s*/*ℓ* interfaces constrained at steady-state. Examples are given of energy fields that autonomously appear on 2-D periodic grain boundary grooves (GBGs). The shapes of these interfaces were obtained analytically from variational theory. Variational GBGs have sharp profiles that allow exact analytic calculations of their curvature distribution, thermochemical potentials, interface gradients, and surface Laplacians, the latter of which represent *inert* divergences of their potential-gradients that do not produce flux responses on sharp interfaces. Microstructure images simulated at steady-state using multiphase-field modeling produce virtually identically shaped diffuse interfaces. Simulated GBG images actually consist of phase-index isolines, which more realistically model the "diffuse” transition between solid and liquid phases. Diffuse interfaces, in contrast to sharp interfaces, allow their curved isolines to develop thermodynamically *active* temperature gradients that drive divergent interfacial heat fluxes. In the case of GBGs, divergent heat fluxes result in interface cooling, which could affect their subsequent growth and pattern formation during solidification.

• Analytic GBGs are ascribed well-posed, well-understood, thermodynamic fields that can’t be measured, but can be calculated. *Calculated* fields for such profiles, of course, evoke neither a past, nor a future, just an instantaneously formed, enduring steady-state. Importantly, comparable thermochemical potentials, their gradients and divergences, predicted for sharp-interface profiles should, in principle, co-develop with real, or even simulated, diffuse-interfaces. In fact, we find simulated GBGs dynamically develop interfacial temperature changes until their steady-state microstructure is reached. These particular phase re-arrangements may, in practice, be observed and measured in the laboratory, or, as demonstrated here, simulated numerically. Where it was possible to establish curved interfaces at steady-state—as shown in the case of periodic GBGs—precision measurements of their interfacial temperature and, indirectly, their divergent flux cooling, become possible through post-processing of the simulated image. See Figs. 4 and 6. In contrast with sharp-interfaces that instantly appear at steady-state, real, or simulated, microstructures arrive, respectively, by thermodynamic evolution from some initial state, or via mathematically described step-wise thermochemical processes. Precision temperature data reported in Fig. 6, using a post-processing algorithm, yield non-linear residuals that are proportionate to the intensity of the isoline’s cooling rate, or the variational surface Laplacian of the interface potential. These data are also supported by companion temperature and heat-flow maps shown in Figs. 7 and 8, that help demonstrate the presence of significant capillary-mediated interfacial cooling.

• Specifically, we showed that divergent heat fluxes, proportional to the surface Laplacian of an interface’s thermochemical potential, also result in gradient divergence equivalent to a distribution of autonomous heat-sinks. Heat-sinks that develop along the *s*/*ℓ* interface cool the surrounding phases by thermal conduction. Capillary-mediated sinks in pure systems modulate the local temperature by only a few tens of milli-Kelvins, and whilst small, these temperature shifts perturb the interface region by generating substantial thermal gradients and cooling. These capillary conduction fields are not confined to the interface itself, but penetrate the spaces of the surrounding bulk crystalline and melt phases. This entire thermodynamic scenario may have significant, and potentially controllable, consequences on microstructure pattern formation during solidification and crystal growth.

• With reference to the predicted distribution of the surface Laplacian and cooling along the sharp-interface counterpart profile of the phase-field image in Fig. 6, one also sees resultant distortion of the isotherms near the *s*/*ℓ* interface, as simulated in Fig. 7. The coldest areas, as predicted, show undercooled "blue” regions that surround each triple junction. A slightly warmer zone, with downward heat flow, extends above the interface. Figure 8 shows the integrated capillary cooling along this stationary *s*/*ℓ* interface appears capable of reversing the mean direction of heat flow below the level of a GBG’s triple points. Solid and liquid phases such as these, with identical bulk thermal conductivities, but lacking capillary cooling, should, to the contrary, have isotherms that remain unaffected by the interface shape. Isotherms without interfacial cooling would appear uniform and straight, as suggested in Fig. 2, unless, of course, active cooling were present, as is predicted here.

• Comparing thermodynamic fields on sharp-interfaces with those on simulated *s*/*ℓ* microstructures with diffuse interfaces can deepen our understanding of processes that subtly control pattern formation during solidification and crystal growth. Although thermodynamic fields determined with both diffuse and sharp interfaces show acceptable agreement between their respective measurement and calculation, there remains a dearth—indeed a void—of experimental data. The authors suggest that advanced facilities and the excellent microgravity environment available on NASA’s International Space Station Laboratory could provide unique opportunities and research support needed to confirm an experimental basis for these novel interfacial phenomena. As capillary-mediated self-interactions result from deterministic physics, they could prove usefully controllable via chemical or physical means, such as adding surfactant elements, applying pressure or magnetic fields^32^. Such practical applications could improve microstructure control in advanced manufacturing based on solidification and crystal growth.

## Data Availability

The datasets generated and/or analyzed during the current study are available from the corresponding author upon reasonable request.

## Code availability

Requests for the post-processing codes used for measurement of interfacial thermochemical residuals may be sent to author Dr. K. Ankit.
